# Multiscale Thermoelastic Analysis of the Thermal Expansion Coefficient and of Microscopic Thermal Stresses of Mature Concrete

**DOI:** 10.3390/ma12172689

**Published:** 2019-08-22

**Authors:** Hui Wang, Herbert Mang, Yong Yuan, Bernhard L. A. Pichler

**Affiliations:** 1 College of Civil Engineering, Tongji University, Shanghai 200092, China; 2 Institute for Mechanics of Materials and Structures, TU Wien—Vienna University of Technology, Karlsplatz 13/202, 1040 Vienna, Austria; 3 State Key Laboratory of Disaster Reduction in Civil Engineering, Tongji University, Shanghai 200092, China

**Keywords:** concrete, thermal expansion, microstresses, thermoelastic, temperature

## Abstract

The thermal expansion coefficient and the microscopic thermal stresses of mature concrete depend on its microstructural composition and the internal relative humidity. This dependence is determined by means of thermoelastic multiscale analysis of concrete. The underlying multiscale model enables two types of scale transition. Firstly, bottom-up homogenization allows for the quantification of the thermal expansion coefficient and the elastic stiffness of concrete based on the Mori-Tanaka scheme. Secondly, top-down scale concentration gives access to the volume averaged stresses experienced by the cement paste, the fine and the coarse aggregates and, furthermore, to the stress states of the interfacial transition zones covering the aggregates. The proposed model is validated by comparing the predicted thermal expansion coefficient of concrete with independent sets of experimental measurements. Finally, sensitivity analyses are carried out to evaluate the influence of the volumetric composition and the internal relative humidity of concrete on the thermal expansion coefficient and the microscopic thermal stresses.

## 1. Introduction

**Figure 1:**
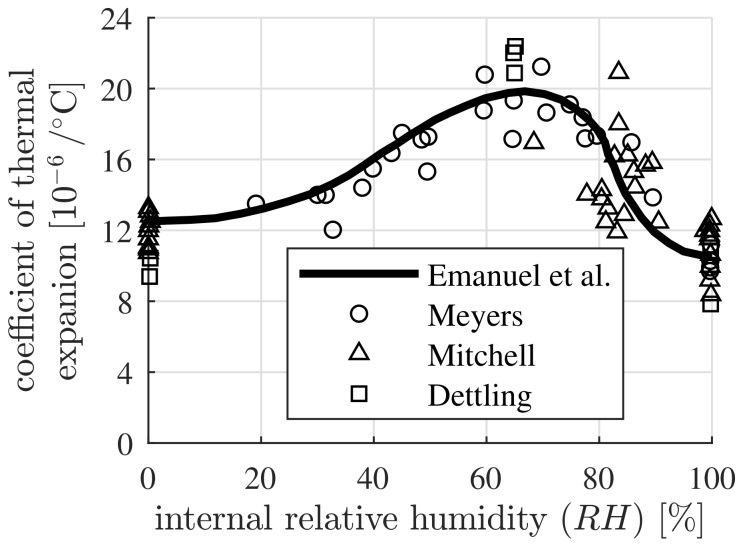
Dependence of the thermal expansion coefficient on the internal relative humidity: experimental data from Meyers [8], Mitchell [9] and Dettling [10], measured on mature cement paste with the initial water-to-cement mass ratio ranging between 0.12 and 0.40; the curve was depicted by Emanuel and Hulsey [1].

The thermal expansion coefficient of mature concrete depends on the aggregate type, the volume fractions of the constituents, the age and the internal relative humidity [1]. The type and the volume fraction of coarse aggregates were shown to be particularly important for the values of the thermal expansion coefficients of concrete [1,2,3]. Aggregates with a rich content of quartz have larger thermal expansion coefficients compared to the ones with a rich content of calcite. Consequently, concrete containing siliceous aggregates exhibits a larger thermal expansion coefficient than that with marble aggregates [4,5]. A linear relation between the thermal expansion coefficient of concrete and the volume fraction of coarse aggregates was reported by Chung [6] and Won [7], respectively. As for the internal relative humidity (RH), the thermal expansion coefficient of concrete becomes a maximum at around RH=60% and a minimum at RH=100% [4,6]. This follows from the dependence of the thermal expansion coefficient of the cement paste on the internal relative humidity, see Figure 1 [1,8,9,10]. This coefficient becomes a minimum value for a fully-saturated (RH=100%) cement paste and it is slightly larger for a fully-dried (RH=0%) cement paste. The thermal expansion coefficient of a partially-saturated cement paste becomes larger. It reaches its maximum value at an internal relative humidity around 65%, which is almost twice of its minimum value [11]. This dependence was recently found to originate from the water uptake/release of the nanosized hydration products at decreasing/increasing temperature [11,12]. In this work, the influence of the composition and the internal relative humidity of concrete on the thermal expansion coefficient will be quantified.

As for quantification of the thermal expansion coefficient of concrete, empirical models, based on the rule of mixtures, have often been used [1,5]. Zhou et al. [13,14] developed a micromechanical model to predict the thermal expansion of concrete and found that the aggregate type was the decisive parameter for the thermal expansion coefficient of concrete while the influence of the initial water-to-cement mass ratio was insignificant. By utilizing micromechanical modeling and a homogenization method, Ghabezloo [15] investigated the effect of the porosity on the thermal expansion coefficient of heterogeneous materials. He concluded that this coefficient may either increase or decrease with increasing porosity, depending on the combination of the thermomechanical properties of the constituents. Liu et al. [16] proposed a stochastic multiscale model for determination of the thermal expansion coefficient of early-age mortar and quantified the significant influence of the type and the volume fraction of the aggregates on this coefficient. By resolving the microstructure of the cement paste to nanosized hydration products and gel pores, the thermoelastic properties of the cement paste were homogenized by means of a microporomechanical model [11]. The present paper refers to upscaling the thermoelastic properties of concrete, from the scale of observation of the cement paste and the fine aggregates, via the intermediate scale of the mortar and the coarse aggregates, to the scale of observation of the homogenized concrete.

Furthermore, microscopically inhomogeneous thermal deformations occur at temperature variations as a result of the heterogeneity of concrete. This leads to microscopic thermal stresses and to microcracking [17,18,19,20,21]. Based on multiscale postprocessing of the macroscopic thermomechanical simulations, Wang et al. [22] concluded that the microstructural stress fluctuations of the cement paste and the aggregates originate from the inhomogeneous thermal expansion and stiffness of the microstructural constituents of concrete. Sumarac and Krasulja [18] investigated the damage of plain concrete due to thermal incompatibility of its constituents. They concluded that a moderate temperature change may cause a substantial degradation of concrete in consequence of microcracking at the interface between the aggregates and the cement paste. By observing the crack propagation with the scanning electron microscope (SEM) and experimentally determining the temperature-dependent stress-strain relation of cement-based materials, Fu et al. [19] concluded that a mismatch of the thermal deformation between the cement paste and the aggregates contributed to the thermal damage of mortar subjected to high temperature. Additionally, numerical simulations with a 2-D mesoscopic thermoelastic damage model have shown that thermal-induced stresses and associated cracking of cement-based composites at elevated temperatures are dominated by the thermal mismatch of the matrix and the inclusions and also by the arrangement, the heterogeneity and the shape of the inclusions [20,21]. This has provided the motivation for the present paper to explicitly quantify the microscopic stresses of the constituents of concrete subjected to thermomechanical loading.

In the present paper, a thermoelastic multiscale model is established to study the macroscopic thermal expansion and the microscopic thermal stresses of concrete in case of temperature changes. To this end, the cement paste, the fine aggregates, the coarse aggregates and the interfacial transition zones are introduced as elementary constituents of concrete. The scale transition from properties of microstructural compositions to macroscopic thermoelastic properties of concrete is carried out by means of the Mori-Tanaka scheme. As for the volume averaged microstresses of the cement paste and the aggregates, the contributions of the macroscopic strains and of the microscopic eigenstrains are considered. At a finer scale of observation, the continuity conditions of both the tractions and displacements across the interfaces, together with the generalized Hooke’s law of the material, give access to the microscopic stress states of the interfacial transition zones surrounding the aggregates.

The paper is structured as follows: The thermoelastic multiscale model of concrete is described in Section 2, followed by its validation with independent sets of experimental data, in Section 3. The influence of the type and the volume fractions of the aggregates and of the internal relative humidity is studied in the framework of sensitivity analysis, in Section 4. Concluding remarks are given in Section 5.

## 2. Thermoelastic Multiscale Model of Concrete

### 2.1. Micromechanical Representation of Concrete

In continuum micromechanics a composite material is considered to be macro-homogeneous but micro-heterogeneous, occupying a representative volume element (RVE) with the characteristic size *ℓ*. It satisfies the separation of scales principle [23]:

(1) d≪ℓ≪D,

*d* denotes the characteristic length of inhomogeneities within the RVE and D stands for the dimension of the structure, made up with this RVE or for the characteristic length of the applied external loading. The ratio ℓ/d is typically in the range of 2 to 3 whereas the ratio D/ℓ typically ranges between 5 to 50 [24,25,26].

In general, the microstructure of RVEs of concrete are too complex to be resolved in full detail. Therefore, instead of it, quasi-homogeneous subdomains are introduced. They are referred to as material phases. The homogenized properties of the RVEs are governed by the properties of the material phases, their shapes, volume fractions and interactions [23].

**Figure 2:**
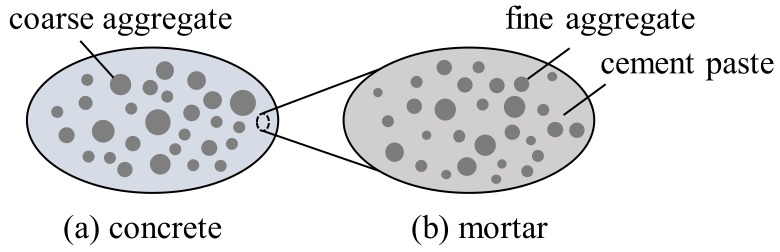
Material organogram of concrete in the form of two-dimensional sketches of three-dimensional representative volume elements: (**a**) concrete consisting of coarse aggregate inclusions, embedded in a mortar matrix, and (**b**) mortar consisting of fine aggregate inclusions, embedded in a cement paste matrix.

The hierarchical organization of concrete, consisting of cement paste, fine aggregates and coarse aggregates, is considered by means of two matrix-inclusion composites, introduced at two different scales of observation. Concrete is idealized as a continuous mortar matrix, hosting spherical coarse aggregate inclusions, see Figure 2a. Mortar is idealized as a continuous cement paste matrix with spherical fine aggregates, embedded as inclusions, see Figure 2b.

### 2.2. Bottom-Up Homogenization of the Macroscopic Thermoelastic Properties

The Mori-Tanaka scheme is well-suited for such matrix-inclusion composites. It allows for the analytical homogenization of the thermoelastic properties of the composites. Each representative volume element (RVE), occupying the domain $V_{RVE}$, is subdivided into a matrix phase and an inclusion phase, occupying the domains $V_{m}$ and $V_{i}$, respectively. Each material phase p∈[m,i] exhibits a specific elastic stiffness $C_{p}$ and a specific eigenstress $\sigma_{p}^{e}$,

(2) $$\forall \underline{x}\in V_{p}\;:\;\left\{\begin{array}{c} C\left(\underline{x}\right)=C_{p}\\ \sigma^{e}\left(\underline{x}\right)=\sigma_{p}^{e} \end{array}\right.,\;p\in \left[\;m\;,\;i\;\right]\;,$$

with a known volume fraction, defined as $f_{p}=V_{p}/V_{RVE}$.

Isotropic elasticity is considered for both the matrix and the inclusion phases. Their elastic stiffness tensors $C_{p}$ are expressed in terms of their bulk moduli $k_{p}$ and shear moduli $\mu_{p}$:

(3) $$C_{p}=3\;k_{p}\;I_{vol}+2\;\mu_{p}\;I_{dev}\;,\;p\in \left[\;m\;,\;i\;\right]\;,$$

where $I_{dev}$ stands for the deviatoric part of the fourth-order unity tensor, defined as $I_{dev}=I-I_{vol}$, where I denotes the symmetric fourth-order unity tensor with $I_{ijrs}=1/2\left(\delta_{ir}\delta_{js}+\delta_{is}\delta_{jr}\right)$ as its components and $I_{vol}=1/3\left(1⊗1\right)$ stands for the volumetric part of the fourth-order unity tensor, where 1 denotes the second-order unity tensor, with the Kronecker delta $\delta_{ij}$ as its components, that is, $\delta_{ij}=1$ for i=j and $\delta_{ij}=0$ otherwise.

The eigenstresses $\sigma_{p}^{e}$ are proportional to the corresponding thermal eigenstrains $\varepsilon_{p}^{e}$

(4) $$\sigma_{p}^{e}=-C_{p}:\varepsilon_{p}^{e}\;,\;p\in \left[\;m\;,\;i\;\right]\;,$$

the thermal eigenstrains are induced by the thermal expansion (or contraction) of the matrix and inclusion phases as the temperature increases (or decreases) by ΔT, that is,

(5) $$\varepsilon_{p}^{e}=\alpha_{p}\;\Delta T\;1\;,\;p\in \left[\;m\;,\;i\;\right]\;,$$

where $\alpha_{m}$ and $\alpha_{i}$ are the thermal expansion coefficients of the matrix and of the inclusion phase, respectively.

The volume fractions of the cement paste, the fine aggregates and the coarse aggregates are obtained from the initial composition of concrete as

(6) $$\begin{array}{cc} f_{cp} & =\frac{\frac{m_{H_{2}O}}{\rho_{H_{2}O}}+\frac{m_{clin}}{\rho_{clin}}}{\frac{m_{H_{2}O}}{\rho_{H_{2}O}}+\frac{m_{clin}}{\rho_{clin}}+\frac{m_{fagg}}{\rho_{fagg}}+\frac{m_{cagg}}{\rho_{cagg}}}\;, \end{array}$$

(7) $$\begin{array}{cc} f_{fagg} & =\frac{\frac{m_{fagg}}{\rho_{fagg}}}{\frac{m_{H_{2}O}}{\rho_{H_{2}O}}+\frac{m_{clin}}{\rho_{clin}}+\frac{m_{fagg}}{\rho_{fagg}}+\frac{m_{cagg}}{\rho_{cagg}}}\;, \end{array}$$

(8) $$\begin{array}{cc} f_{cagg} & =\frac{\frac{m_{cagg}}{\rho_{cagg}}}{\frac{m_{H_{2}O}}{\rho_{H_{2}O}}+\frac{m_{clin}}{\rho_{clin}}+\frac{m_{fagg}}{\rho_{fagg}}+\frac{m_{cagg}}{\rho_{cagg}}}\;, \end{array}$$

in these relations, $m_{H_{2}O}$, $m_{clin}$, $m_{fagg}$ and $m_{cagg}$ denote the mass of water and of the cement clinkers, the fine aggregates and the coarse aggregates per cubic meter of the concrete, while $\rho_{H_{2}O}$, $\rho_{clin}$, $\rho_{fagg}$ and $\rho_{cagg}$ stand for their mass densities. Notably, these values are fractions of the overall volume of concrete. Hence, their sum is equal to 1:

(9) $$f_{cp}+f_{fagg}+f_{cagg}=1\;,$$

at the scale of the mortar, the matrix is the cement paste and the inclusions are the fine aggregates. The mortar-related volume fractions of the matrix and the inclusion phases read as

(10) $$f_{m}^{mort}=\frac{f_{cp}}{f_{cp}+f_{fagg}}\;,\;f_{i}^{mort}=1-f_{m}^{mort}\;,$$

at the scale of the concrete, the matrix is the mortar and the inclusions are the coarse aggregates. The concrete-related volume fractions of the matrix and the inclusion phases read as

(11) $$f_{m}^{con}=f_{cp}+f_{fagg}\;,\;f_{i}^{con}=1-f_{m}^{con}\;.$$

The generalized Hooke’s law is used for the homogenized matrix-inclusion composite. It results in

(12) $$\sum_{\mathrm{hom}}=C_{\mathrm{hom}}:\left(E_{\mathrm{hom}}-E_{\mathrm{hom}}^{e}\right)=C_{\mathrm{hom}}:E_{\mathrm{hom}}+\sum_{\mathrm{hom}}^{e}\;,$$

where $\sum_{\mathrm{hom}}$ and $E_{\mathrm{hom}}$ represent the macroscopic stress and strain, respectively. The homogenized stiffness tensor reads as [27]

(13) $$C_{\mathrm{hom}}=f_{m}\;C_{m}:A_{m}+f_{i}\;C_{i}:A_{i}$$

and the homogenized eigenstress as [22,28]

(14) $$\sum_{\mathrm{hom}}^{e}=f_{m}\;\sigma_{m}^{e}:A_{m}+f_{i}\;\sigma_{i}^{e}:A_{i}\;,$$

where $A_{m}$ and $A_{i}$ denote the strain concentration tensors of the matrix and the inclusion phase. They can be estimated by means of the Mori-Tanaka scheme as [23,29]

(15) $$A_{p}={\left[I+S:C_{m}^{-1}:\left(C_{p}-C_{m}\right)\right]}^{-1}:{\left\{\sum_{\;j=m,i}f_{j}{\left[I+S:C_{m}^{-1}:\left(C_{j}-C_{m}\right)\right]}^{-1}\right\}}^{-1}\;,\;\;p\in \left[\;m\;,\;i\;\right]\;,$$

where S is the Eshelby tensor of a spherical inclusion, embedded in an infinite matrix of stiffness $C_{m}$. It is defined as [30]

(16) $$S=S_{vol}\;I_{vol}+S_{dev}\;I_{dev}\;,$$

with

(17) $$S_{vol}=\frac{3\;k_{m}}{3\;k_{m}+4\;\mu_{m}}$$

and

(18) $$S_{dev}=\frac{6\;(k_{m}+2\;\mu_{m})}{5\;(3\;k_{m}+4\;\mu_{m})}\;,$$

inserting Equation (16) into Equation (15) results in the strain concentration tensor

(19) $$A_{p}=A_{p,vol}\;I_{vol}+A_{p,dev}\;I_{dev}\;,\;p\in \left[\;m\;,\;i\;\right]\;,$$

with

(20) $$A_{p,vol}={\left(1+S_{vol}\frac{k_{p}-k_{m}}{k_{m}}\right)}^{-1}{\left[\sum_{j=m,i}f_{j}{\left(1+S_{vol}\frac{k_{j}-k_{m}}{k_{m}}\right)}^{-1}\right]}^{-1}\;$$

and

(21) $$A_{p,dev}={\left(1+S_{dev}\frac{\mu_{p}-\mu_{m}}{\mu_{m}}\right)}^{-1}{\left[\sum_{j=m,i}f_{j}{\left(1+S_{dev}\frac{\mu_{j}-\mu_{m}}{\mu_{m}}\right)}^{-1}\right]}^{-1}\;,$$

where $A_{p,vol}$ and $A_{p,dev}$ denote the volumetric and the deviatoric part of the strain concentration tensor, respectively. The homogenized stiffness tensor is obtained from inserting Equation (16) into Equation (15), followed by substituting the result together with Equation (3) into Equation (13):

(22) $$C_{\mathrm{hom}}=3\;k_{\mathrm{hom}}\;I_{vol}+2\;\mu_{\mathrm{hom}}\;I_{dev}\;,$$

$k_{\mathrm{hom}}$ and $\mu_{\mathrm{hom}}$ stand for the homogenized bulk and shear modulus, respectively, of the composite, that is,

(23) $$k_{\mathrm{hom}}=\frac{f_{i}\;k_{i}{\left[1+\frac{S_{vol}\;\left(k_{i}-k_{m}\right)}{k_{m}}\right]}^{-1}+f_{m}\;k_{m}}{f_{i}{\left[1+\frac{S_{vol}\;\left(k_{i}-k_{m}\right)}{k_{m}}\right]}^{-1}+f_{m}}\;,$$

and

(24) $$\mu_{\mathrm{hom}}=\frac{f_{i}\;\mu_{i}{\left[1+\frac{S_{dev}\;\left(\mu_{i}-\mu_{m}\right)}{\mu_{m}}\right]}^{-1}+f_{m}\;\mu_{m}}{f_{i}{\left[1+\frac{S_{dev}\;\left(\mu_{i}-\mu_{m}\right)}{\mu_{m}}\right]}^{-1}+f_{m}}\;.$$

As for quantifying the thermal expansion coefficient of the homogenized composite, $\alpha_{\mathrm{hom}}$, the composite is assumed to deform freely. Hence, the macroscopic stress tensor vanishes. For this scenario, Equation (12) yields

(25) $$\sum_{\mathrm{hom}}=0\;\Rightarrow \;E_{\mathrm{hom}}=E_{\mathrm{hom}}^{e}=-C_{\mathrm{hom}}^{-1}:\sum_{\mathrm{hom}}^{e}\;,$$

the sought expression for $\alpha_{\mathrm{hom}}$ is obtained by writing the homogenized eigenstrain $E_{\mathrm{hom}}^{e}$ in Equation (25) as $\alpha_{\mathrm{hom}}\Delta T1$ and inserting Equation (5) into Equation (4), followed by substituting the obtained expression into Equation (14) and, finally, by inserting the outcome together with Equation (13) into Equation (25). After division by ΔT, this yields [22]

(26) $$\alpha_{\mathrm{hom}}\;1={\left[f_{m}C_{m}:A_{m}+f_{i}C_{i}:A_{i}\right]}^{-1}:\left[\alpha_{m}f_{m}\left(C_{m}:1\right):A_{m}+\alpha_{i}f_{i}\left(C_{i}:1\right):A_{i}\right]\;,$$

inserting Equations (20) and (21) into Equation (19) and substituting the result into Equation (26) provides the analytical solution of the thermal expansion coefficient of the homogenized matrix-inclusion composite:

(27) $$\alpha_{\mathrm{hom}}=\frac{3\;k_{i}\;k_{m}\;\left(\alpha_{m}\;f_{m}+\alpha_{i}\;f_{i}\right)+4\;\mu_{m}\;\left(\alpha_{m}\;f_{m}\;k_{m}+\alpha_{i}\;f_{i}\;k_{i}\right)}{3\;k_{i}\;k_{m}+4\;\mu_{m}\;\left(f_{m}\;k_{m}+f_{i}\;k_{i}\right)}\;.$$

Homogenization of the concrete is carried out in two steps. Firstly, the matrix-inclusion composite of mortar in Figure 2b is homogenized. The stiffness tensor and the thermal expansion coefficient of mortar are calculated by inserting the quantities that stand for material properties of the cement paste matrix and of the fine aggregate inclusions into Equations (13) and (27). The resulting solutions serve as input for the subsequent homogenization of the matrix-inclusion composite of concrete in Figure 2a in the second step. The stiffness tensor and the thermal expansion coefficient of concrete are computed by inserting the quantities that represent material properties of the homogenized mortar matrix and of the coarse aggregate inclusions into Equations (13) and (27).

### 2.3. Top-Down Quantification of the Microscopic Thermal Stresses

#### 2.3.1. Volume Averaged Stresses of the Constituents of Concrete

The volume averaged microstresses of the matrix and the inclusions are quantified by top-down scale transition, based on knowledge of the macrostress tensor $\sum_{\mathrm{hom}}$ and of the temperature change ΔT. The homogenized eigenstress $\sum_{\mathrm{hom}}^{e}$ is quantified by substituting Equation (5) into Equation (4) and inserting the result into Equation (14). Substituting the latter together with Equation (13) into Equation (12) allows for quantification of the macroscopic strain $E_{\mathrm{hom}}$ as

(28) $$E_{\mathrm{hom}}=C_{\mathrm{hom}}^{-1}:\left(\sum_{\mathrm{hom}}-\sum_{\mathrm{hom}}^{e}\right)\;,$$

considering the influence of the macroscopic strain $E_{\mathrm{hom}}$ and of the microscopic eigenstrain $\varepsilon_{p}^{e}$, the volume averaged microscopic strain $\varepsilon_{p}$ is expressed as [31,32]

(29) $$\varepsilon_{p}=A_{p}:E_{\mathrm{hom}}+\sum_{q=m,i}D_{pq}:\varepsilon_{q}^{e}\;,\;p\in \left[\;m\;,\;i\;\right]\;,$$

where $D_{pq}$ stands for the eigenstrain influence tensor. For q=p, it expresses the influence of the eigenstrain of phase *p* on its the microstrain, reading as [31]

(30) $$D_{pp}=\left[I-f_{p}A_{p}\right]:{\left[I+S:C_{m}^{-1}:\left(C_{p}-C_{m}\right)\right]}^{-1}:\left(S:C_{m}^{-1}\right):C_{p}\;,$$

for q≠p, it expresses the influence of the eigenstrain of phase *q* on the microstrain of phase *p*, reading as [31]

(31) $$D_{pq}=-A_{p}:f_{q}{\left[I+S:C_{m}^{-1}:\left(C_{q}-C_{m}\right)\right]}^{-1}:\left(S:C_{m}^{-1}\right):C_{q}\;,$$

considering Equations (3) and (19), the eigenstrain influence tensors may be split into a volumetric and a deviatoric part, that is,

(32) $$\begin{array}{c} D_{pp}=D_{pp,vol}\;I_{vol}+D_{pp,dev}\;I_{dev}\;,\\ D_{pq}=D_{pq,vol}\;I_{vol}+D_{pq,dev}\;I_{dev}\;, \end{array}$$

inserting Equation (32) into Equations (30) and (31) and substituting the result together with Equation (5) into Equation (29) delivers the volume averaged microscopic strains of the matrix and of the inclusions as

(33) $$\begin{array}{c} \varepsilon_{m}=A_{m}:E_{\mathrm{hom}}+D_{mm,vol}\;\alpha_{m}\;\Delta T\;1+D_{mi,vol}\;\alpha_{i}\;\Delta T\;1\;,\\ \varepsilon_{i}=A_{i}:E_{\mathrm{hom}}+D_{im,vol}\;\alpha_{m}\;\Delta T\;1+D_{ii,vol}\;\alpha_{i}\;\Delta T\;1\;, \end{array}$$

with

(34) $$\begin{array}{c} D_{mm,vol}=\left(1-f_{m}\;A_{m,vol}\right)\;S_{vol}\;,\\ D_{mi,vol}=-f_{i}\;A_{m,vol}{\left(1+S_{vol}\;\frac{k_{i}-k_{m}}{k_{m}}\right)}^{-1}\;S_{vol}\;\frac{k_{i}}{k_{m}}\;,\\ D_{im,vol}=-f_{m}\;A_{i,vol}\;S_{vol}\;,\\ D_{ii,vol}=\left(1-f_{i}\;A_{i,vol}\right){\left(1+S_{vol}\;\frac{k_{i}-k_{m}}{k_{m}}\right)}^{-1}\;S_{vol}\;\frac{k_{i}}{k_{m}}\;, \end{array}$$

The volume averaged microstresses of the matrix and inclusion phases, $\sigma_{m}$ and $\sigma_{i}$, respectively, finally follow from the elasticity law as

(35) $$\sigma_{m}=C_{m}:\left(\varepsilon_{m}-\varepsilon_{m}^{e}\right)\;,\;\sigma_{i}=C_{i}:\left(\varepsilon_{i}-\varepsilon_{i}^{e}\right)\;.$$

Top-down computation of the microstresses with the help of the Equations (28)–(35) is first performed for the representative volume element of concrete, see Figure 2a, based on knowledge of the macroscopic stresses of concrete and of the temperature fields obtained from structural simulations. This gives access to the volume averaged stresses of the mortar and of the coarse aggregates. Thereafter, this top-down analysis is applied to the representative volume element of mortar, see Figure 2b, taking the computed stress state of mortar as input for quantifying the volume averaged stresses of the cement paste and the fine aggregates. Notably, given the fact that the thickness of the interfacial transition zones is much smaller than the dimensions of the aggregate inclusions, they are idealized as two-dimensional interfaces, representing a firm bond between the matrix phase and the inclusion phases.

#### 2.3.2. Microstress States of the Interfacial Transition Zones

Interfacial transition zones are thin layers of the *porous* cement paste covering the aggregates. They represent the weakest links in the microstructure of concrete [33]. Given the small volume fraction of ITZs, they are not distinguished from the *bulk* cement paste. Thus, for estimation of the homogenized stiffness and the thermal expansion coefficient of concrete, they are idealized as two-dimensional interfaces. However, microscopic stress states of the ITZs are important for investigation of cracking and of the strength of concrete [26,34]. In order to quantify the microstress states of ITZs, they are resolved as three-dimensional shells covering the aggregates at a finer scale of observation.

Assuming a firm bond between the aggregate inclusions and the surrounding ITZ shells, continuity conditions of both the tractions and displacements must be satisfied across the interfaces. Traction continuity results in the compatibility relation for the stresses of the inclusion, $\sigma_{i}$, and of the ITZ, $\sigma_{ITZ}$, across the interface $I_{i}^{ITZ}$ [34]

(36) $$\left[\sigma_{i}-\sigma_{ITZ}\left(\underline{x}\right)\right]\cdot \underline{n}\left(\underline{x}\right)=0\;,\;\forall \underline{x}\in I_{i}^{ITZ}\;,$$

where $\underline{n}\left(\underline{x}\right)$ represents the outward unit normal vector at the position $\underline{x}$ of the interface. The requirement of displacement continuity results in the compatibility relation for the strains of the inclusion phase, $\varepsilon_{i}$, and of the ITZ, $\varepsilon_{ITZ}$, across the interface $I_{i}^{ITZ}$ [34]

(37) $${\underline{t}}_{1}\left(\underline{x}\right)\cdot \left[\varepsilon_{i}-\varepsilon_{ITZ}\left(\underline{x}\right)\right]\cdot {\underline{t}}_{2}\left(\underline{x}\right)=0\;,\;\forall \underline{x}\in I_{i}^{ITZ}\;,$$

where ${\underline{t}}_{1}\left(\underline{x}\right)$ and ${\underline{t}}_{2}\left(\underline{x}\right)$ denote an arbitrary pair of vectors in the tangential plane of the interface at the position $\underline{x}$. Notably, homogeneous microstrains and microstresses are good approximations of the actual microstress and microstrain states inside the inclusions, as suggested by the Mori-Tanaka estimation [23]. Hence, the volume averaged stress state, $\sigma_{i}$, and the corresponding strain state, $\varepsilon_{i}$, are also representative for the surfaces of the aggregate inclusions, irrespective of the position $\underline{x}$, see Equations (36) and (37).

**Figure 3:**
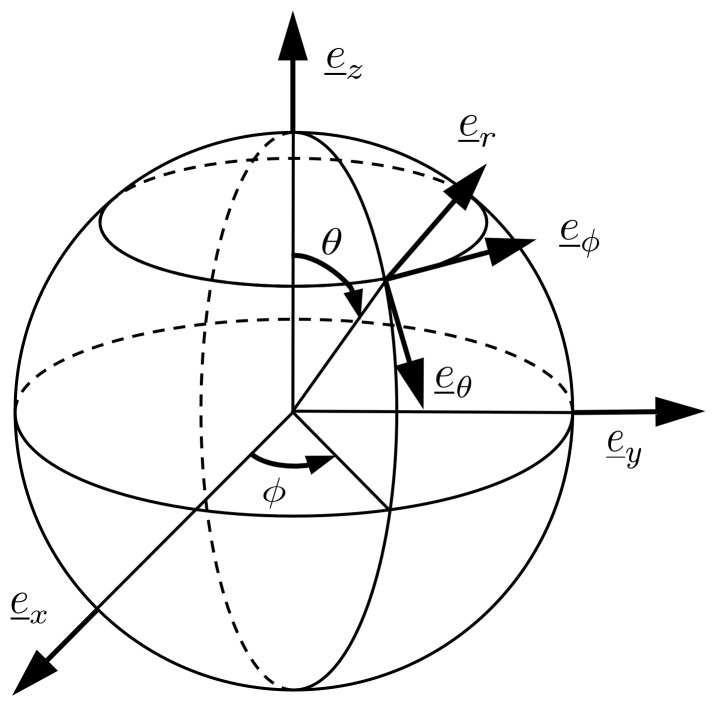
Local spherical coordinate system covering the inclusion phase.

A local spherical coordinate system $e=(e_{r},e_{\theta },e_{\phi })$ is introduced, see Figure 3. The zenith angle θ and the azimuth angle ϕ determine the considered point of the ITZ.

Transformation of the Cartesian components of the stress tensor and the strain tensor into components related to the local spherical coordinate system is carried out by means of the transformation matrix

(38) $$Q=\left(\begin{array}{ccc} \cos\phi \;\sin\theta & \sin\phi \;\sin\theta & \cos\theta \\ \cos\phi \;\cos\theta & \sin\phi \;\cos\theta & -\sin\theta \\ -\sin\phi & \cos\phi & 0 \end{array}\right)\;,$$

the transformation rule for the stress components reads as

(39) $$\left(\begin{array}{ccc} \sigma_{agg,rr} & \sigma_{agg,r\theta } & \sigma_{agg,r\phi }\\ \sigma_{agg,r\theta } & \sigma_{agg,\theta \theta } & \sigma_{agg,\theta \phi }\\ \sigma_{agg,r\phi } & \sigma_{agg,\theta \phi } & \sigma_{agg,\phi \phi } \end{array}\right)=Q\cdot \left(\begin{array}{ccc} \sigma_{agg,xx} & \sigma_{agg,xy} & \sigma_{agg,xz}\\ \sigma_{agg,xy} & \sigma_{agg,yy} & \sigma_{agg,yz}\\ \sigma_{agg,xz} & \sigma_{agg,yz} & \sigma_{agg,zz} \end{array}\right)\cdot Q^{T}\;,$$

where $Q^{T}$ stands for the transpose of Q. Replacing σ by ε in Equation (39) delivers the corresponding transformation rule for the strain components. Substituting the unit outward normal vector $\underline{n}$, reading as (1,0,0) in the local spherical coordinate system, into Equation (36) results in the continuity relations for the three stress components with the index *r* [34]

(40) $$\begin{array}{c} \sigma_{agg,rr}\left(\theta ,\phi \right)=\sigma_{ITZ,rr}\left(\theta ,\phi \right)\;,\\ \sigma_{agg,r\theta }\left(\theta ,\phi \right)=\sigma_{ITZ,r\theta }\left(\theta ,\phi \right)\;,\\ \sigma_{agg,r\phi }\left(\theta ,\phi \right)=\sigma_{ITZ,r\phi }\left(\theta ,\phi \right)\;, \end{array}$$

considering the pair of vectors (${\underline{t}}_{1}$, ${\underline{t}}_{2}$) in Equation (37) as (${\underline{e}}_{\theta }$, ${\underline{e}}_{\theta }$), (${\underline{e}}_{\phi }$, ${\underline{e}}_{\phi }$) and (${\underline{e}}_{\theta }$, ${\underline{e}}_{\phi }$), respectively, yields the continuity relations for the three strain components without the index *r*

(41) $$\begin{array}{c} \varepsilon_{agg,\theta \theta }\left(\theta ,\phi \right)=\varepsilon_{ITZ,\theta \theta }\left(\theta ,\phi \right)\;,\\ \varepsilon_{agg,\phi \phi }\left(\theta ,\phi \right)=\varepsilon_{ITZ,\phi \phi }\left(\theta ,\phi \right)\;,\\ \varepsilon_{agg,\theta \phi }\left(\theta ,\phi \right)=\varepsilon_{ITZ,\theta \phi }\left(\theta ,\phi \right)\;. \end{array}$$

Consideration of the continuity of the tractions and the displacements permits determination of the three stress and the three strain components of the ITZ, see Equations (40) and (41), based on knowledge of the stresses and the strains of the aggregates. The remaining unknown stress and strain components are determined by means of the generalized Hooke’s law for the ITZ, reading as [35]

(42) $$\begin{array}{ccc} \left(\begin{array}{c} \sigma_{ITZ,rr}\\ \sigma_{ITZ,\theta \theta }\\ \sigma_{ITZ,\phi \phi }\\ \sqrt{2}\;\sigma_{ITZ,\theta \phi }\\ \sqrt{2}\;\sigma_{ITZ,r\phi }\\ \sqrt{2}\;\sigma_{ITZ,r\theta } \end{array}\right) & = & \left(\begin{array}{cccccc} k_{ITZ}+\frac{4}{3}\mu_{ITZ} & k_{ITZ}-\frac{2}{3}\mu_{ITZ} & k_{ITZ}-\frac{2}{3}\mu_{ITZ} & 0 & 0 & 0\\ k_{ITZ}-\frac{2}{3}\mu_{ITZ} & k_{ITZ}+\frac{4}{3}\mu_{ITZ} & k_{ITZ}-\frac{2}{3}\mu_{ITZ} & 0 & 0 & 0\\ k_{ITZ}-\frac{2}{3}\mu_{ITZ} & k_{ITZ}-\frac{2}{3}\mu_{ITZ} & k_{ITZ}+\frac{4}{3}\mu_{ITZ} & 0 & 0 & 0\\ 0 & 0 & 0 & 2\;\mu_{ITZ} & 0 & 0\\ 0 & 0 & 0 & 0 & 2\;\mu_{ITZ} & 0\\ 0 & 0 & 0 & 0 & 0 & 2\;\mu_{ITZ} \end{array}\right)\\ & & \cdot \left(\begin{array}{c} \varepsilon_{ITZ,rr}\\ \varepsilon_{ITZ,\theta \theta }\\ \varepsilon_{ITZ,\phi \phi }\\ \sqrt{2}\;\varepsilon_{ITZ,\theta \phi }\\ \sqrt{2}\;\varepsilon_{ITZ,r\phi }\\ \sqrt{2}\;\varepsilon_{ITZ,r\theta } \end{array}\right)\;-\;3\;k_{ITZ}\;\left(\begin{array}{c} \alpha_{ITZ}\;\Delta T\\ \alpha_{ITZ}\;\Delta T\\ \alpha_{ITZ}\;\Delta T\\ 0\\ 0\\ 0 \end{array}\right), \end{array}$$

where $k_{ITZ}$ and $\mu_{ITZ}$ denote the bulk and the shear modulus of the ITZ, respectively, and $\alpha_{ITZ}$ stands for the thermal expansion coefficient of the ITZ. The sought three stress components read as

(43) $$\begin{array}{c} \begin{array}{cc} \sigma_{ITZ,\theta \theta }\left(\theta ,\phi \right)= & \;[4\;\mu_{ITZ}\;\left(3\;k_{ITZ}+\mu_{ITZ}\right)\;\varepsilon_{ITZ,\theta \theta }+\left(3\;k_{ITZ}-2\;\mu_{ITZ}\right)\;\left(2\;\mu_{ITZ}\;\varepsilon_{ITZ,\phi \phi }+\sigma_{ITZ,rr}\right)\\ & -18\;k_{ITZ}\mu_{ITZ}\;\alpha_{ITZ}\;\Delta T]/\left(3\;k_{ITZ}+4\;\mu_{ITZ}\right)\;, \end{array}\\ \begin{array}{cc} \sigma_{ITZ,\phi \phi }\left(\theta ,\phi \right)= & \;[4\;\mu_{ITZ}\;\left(3\;k_{ITZ}+\mu_{ITZ}\right)\;\varepsilon_{ITZ,\phi \phi }+\left(3\;k_{ITZ}-2\;\mu_{ITZ}\right)\;\left(2\;\mu_{ITZ}\;\varepsilon_{ITZ,\theta \theta }+\sigma_{ITZ,rr}\right)\\ & -18\;k_{ITZ}\mu_{ITZ}\;\alpha_{ITZ}\;\Delta T]/\left(3\;k_{ITZ}+4\;\mu_{ITZ}\right)\;, \end{array}\\ \sigma_{ITZ,\theta \phi }\left(\theta ,\phi \right)=2\;\mu_{ITZ}\;\varepsilon_{ITZ,\theta \phi }\;, \end{array}$$

and the sought three strain components as

(44) $$\begin{array}{c} \varepsilon_{ITZ,rr}\left(\theta ,\phi \right)=\frac{3\;\sigma_{ITZ,rr}-\left(3\;k_{ITZ}-2\;\mu_{ITZ}\right)\;\left(\varepsilon_{ITZ,\theta \theta }+\varepsilon_{ITZ,\phi \phi }\right)+9\;k_{ITZ}\;\mu_{ITZ}\alpha_{ITZ}\;\Delta T}{3\;k_{ITZ}+4\;\mu_{ITZ}}\;,\\ \varepsilon_{ITZ,r\theta }\left(\theta ,\phi \right)=\frac{\sigma_{ITZ,r\theta }}{2\;\mu_{ITZ}}\;,\\ \varepsilon_{ITZ,r\phi }\left(\theta ,\phi \right)=\frac{\sigma_{ITZ,r\phi }}{2\;\mu_{ITZ}}\;. \end{array}$$

## 3. Model Validation of the Thermal Expansion Coefficient of Fully-Saturated Concrete

Various test results of the thermal expansion coefficients of mature cementitious materials are available in the open literature. They are significantly influenced by the test set-up. A relatively new test method, recommended by the American Association of State Highway and Transport Officials, AASHTO TP 60 [36], has become widely accepted for determination of thermal expansion coefficients of cementitious materials. It is based on measuring the length change of a fully-saturated cementitious specimen, that is, for RH=100%, due to a specific temperature change. Following this standard method, Sakyi-Bekoe [2], Tasneem et al. [3] and Naik et al. [37] independently determined the thermal expansion coefficients of concretes with different initial compositions experimentally. Their results are used for validation of the proposed thermoelastic model.

### 3.1. Thermoelastic Properties of the Cement Paste and of the Aggregates

Thermoelastic properties of the cement paste and of the aggregates are input for the proposed multiscale model. After mixing the cement clinker with water, the elastic modulus and the strength of the cement paste are growing fast in the first week and then are slowing down. Haecker et al. [38] measured the elastic modulus of the cement paste, made of ASTM type I cement with an initial water-to-cement mass ratio ranging between 0.25 and 0.60, at the age of 14 days and 56 days, respectively, after mixing. The latter, which are slightly larger than the former, are taken as the elastic moduli of mature cement pastes, see Table 1. Poisson’s ratio of the mature cement paste is considered to be constant and equal to 0.20 [13]. The corresponding values of the bulk modulus *k* and the shear modulus μ in Equation (3) can be determined based on the standard relations for isotropic materials,

(45) $$k=\frac{E}{3(1-2\;\nu )}\;,\;\mu =\frac{E}{2(1+\nu )}\;,$$

where *E* and ν denote the elastic modulus and Poisson’s ratio, respectively. The thermal expansion coefficient of the fully-saturated cement paste is set equal to $10.5\times 10^{-6}{/}^{\circ }C$, see Figure 1.

The elastic modulus, Poisson’s ratio and the thermal expansion coefficient of the aggregate depend on the mineral composition, the porosity, the crystal orientation and the texture of the aggregate rock [1,5,39,40]. Thus, the thermoelastic properties of the same type of aggregate from different sources can be different. Emanuel et al. [1] and Gudmundsson [39] listed the range of the thermal expansion coefficients and of the elastic parameters of typical rock aggregates, respectively. Their average values, see Table 2, are taken herein as input for prediction of the thermal expansion coefficient of concrete.

### 3.2. Comparison with Independent Experimental Measurements

(1) Experiments by Sakyi-Bekoe

Sakyi-Bekoe [2] measured the thermal expansion coefficients of concretes from Alabama, USA. Granite, siliceous river gravel and dolomitic limestone, respectively, were used as the coarse aggregates, while siliceous sand was used as the fine aggregate. Three different ratios of volume fraction of the fine and the coarse aggregates were considered, namely, $f_{fagg}/f_{cagg}=\left[\;40/60\;,45/55\;,50/50\;\right]$. Three different values of the initial water-to-cement mass ratio were chosen, namely, w/c=[0.32,0.38,0.44]. As the initial water-to-cement mass ratio w/c increased from 0.32 to 0.44, the volume fraction of the cement paste, $f_{cp}$, decreased from 0.35 to 0.31.

As for the model prediction, the thermoelastic properties of siliceous sand, granite, siliceous river gravel and dolomitic limestone were taken as the ones of quartzite, granite, sandstone and dolomite, from Table 2. The elastic moduli of the cement pastes with different initial water-to-cement mass ratios are obtained by interpolating the values in Table 1. Input for model predictions, namely, volume fractions and thermoelastic properties of the constituents of concrete, are summarized in Table 3a,b.

The model predicted thermal expansion coefficients of concretes with different initial compositions agree well with the experimental measurements, see Table 4. Considering the latter as the reference values, the mean absolute error of this set of model predictions, that is, the deviation from the experimental measurements, is equal to $0.65\times 10^{-6}{/}^{\circ }C$. The root mean square error is equal to $0.84\times 10^{-6}{/}^{\circ }C$. These values are relatively small compared to the reference values.

(2) Experiments by Tasneem et al.

Tasneem et al. [3] measured the thermal expansion coefficients of concretes with different types and volume fractions of aggregates. Granite and dolomite were used as the coarse aggregates while the fine aggregates were natural sand, originated from marine sedimentary rock, and manufactured sand, crushed from granite, respectively. The volume fraction of the cement paste, $f_{cp}$, was set equal to 0.33, with an initial water-to-cement mass ratio w/c of 0.50.

As for the model prediction, the thermoelastic properties of natural sand were taken as the ones of the sandstone. The input thermoelastic properties of the cement paste, the fine aggregates and the coarse aggregates are summarized in Table 5. The volume fractions of the fine and the coarse aggregates, $f_{fagg}$ and $f_{cagg}$, were determined according to the mass mixture and the mass density, see Table 6.

The model predicted thermal expansion coefficients of concretes with different initial compositions agree well with the experimental measurements, see Table 6. Considering the latter as the reference values, the mean absolute error of this set of model predictions, that is, the deviation from the experimental measurements, is equal to $0.28\times 10^{-6}{/}^{\circ }C$. The root mean square error is equal to $0.30\times 10^{-6}{/}^{\circ }C$. These values are relatively small compared to the reference values.

(3) Experiments by Naik et al.

Naik et al. [37] measured the thermal expansion coefficients of concretes with six types of coarse aggregates, namely, glacial gravel, quartzite, granite, diabase, basalt and dolomite. Natural sand was used for the fine aggregates. The initial water-to-cement mass ratio w/c was around 0.40.

As for the model prediction, the thermoelastic properties of glacial gravel and natural sand were taken as the ones of sandstone. The input thermoelastic properties of the cement paste, the fine aggregates and the coarse aggregates are summarized in Table 7. Volume fractions of the cement paste, the fine aggregates and the coarse aggregates follow from the mass mixture and the mass density. They were found to be almost constant, reading as

(46) $$f_{cp}=0.30\;,\;f_{fagg}=0.25\;,\;f_{cagg}=0.45\;.$$

The model predicted thermal expansion coefficients of concretes with different types of coarse aggregates agree well with the experimental measurements, see Table 8. Considering the latter as the reference values, the mean absolute error of this set of model predictions, that is, the deviation from the experimental measurements, is equal to $0.70\times 10^{-6}{/}^{\circ }C$. The root mean square error is equal to $0.74\times 10^{-6}{/}^{\circ }C$. These values are relatively small compared to the reference values.

**Figure 4:**
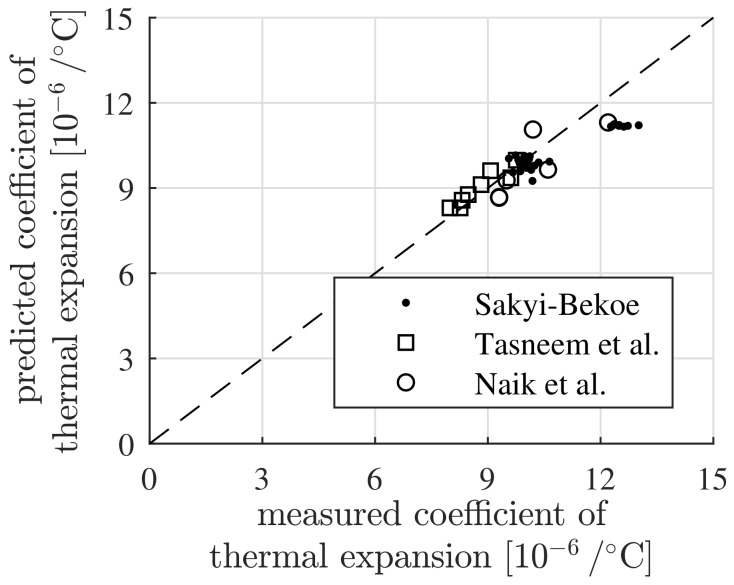
Comparison of model predicted thermal expansion coefficients of concrete with results from experimental measurements by Sakyi-Bekoe [2], Tasneem et al. [3] and Naik et al. [37].

The comparison of model predicted thermal expansion coefficients of concrete with experimentally measured values in Table 4, Table 6 and Table 8 is summarized in Figure 4. It demonstrates the usefulness of the proposed model. Fluctuations of the thermoelastic properties of the aggregates and the cement paste may be the reason of the relatively small differences between the model predictions and the experimental measurements.

## 4. Sensitivity Analysis

The type and the volume fraction of the aggregates, as well as the internal relative humidity, are reported to have a significant influence on the thermal expansion coefficient of concrete [1,2,3,4,6,8,9,10,13,14,37]. By utilizing the established thermoelastic model of concrete, the sensitivity of the thermal expansion coefficient and the thermal microstresses of concrete with respect to the material composition and the internal relative humidity are investigated.

### 4.1. Sensitivity of the Thermal Expansion Coefficient with Respect to the Volumetric Composition of Concrete

The thermal expansion coefficient of concrete, containing three different coarse aggregates, namely, sandstone, granite and limestone, respectively, with different volume fractions, is determined. The initial water-to-cement mass ratio w/c is set equal to 0.40. The internal relative humidity, RH, is assumed as approximately 50%, resulting in $18\times 10^{-6}{/}^{\circ }C$ as the value of the thermal expansion coefficient of the cement paste, see Figure 1. Sandstone is used for the fine aggregates. Thermoelastic properties of the cement paste, the fine aggregates and of three types of coarse aggregates, serving as input, are listed in Table 9.

The volume fraction of the cement paste is a constant, given as $f_{cp}=0.30$. The volume fraction of the coarse aggregates with respect to the total volume of aggregates varies from 0.20 to 0.80:

(47) $$f_{cagg}/\left(f_{fagg}+f_{cagg}\right)\in \left[\;0.20\;,0.30\;,0.40\;,0.50\;,0.60\;,0.70\;,0.80\;\right]\;.$$

**Figure 5:**
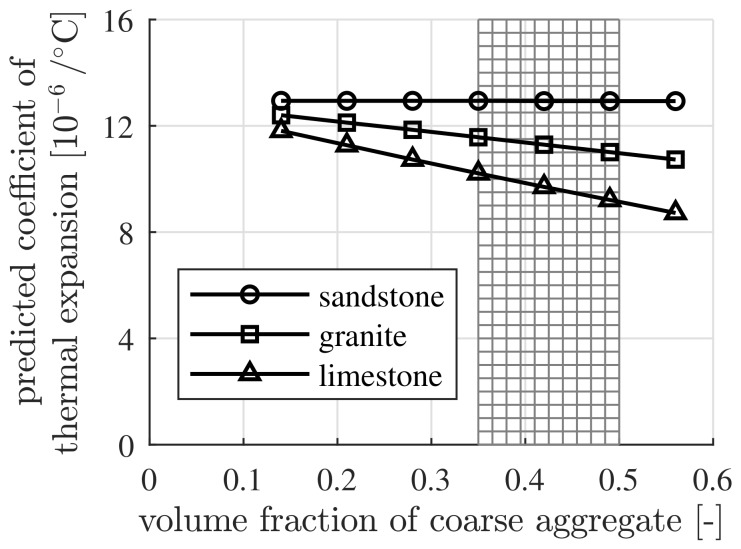
Dependence of the thermal expansion coefficient of concrete on the type and the volume fraction of the coarse aggregates, with RH=50%, $f_{cp}=0.30$ and w/c=0.40.

The computed values of the thermal expansion coefficient of concrete strongly depend on the type and the volume fraction of the coarse aggregates, see Figure 5. For adequate consolidation of the concrete, the volume fraction of the coarse aggregates is generally in the range of 35% to 50% [41], see the shaded area in Figure 5. The mixtures outside this range are just part of a laboratory study. The concrete made with sandstone exhibits the largest thermal expansion coefficient, while that with limestone exhibits the smallest one. The thermal expansion coefficient of concrete depends nearly linearly on the volume fraction of coarse aggregates.

### 4.2. Sensitivity of the Thermal Expansion Coefficient of Concrete with Respect to the Internal Relative Humidity

The thermal expansion coefficient of concrete, containing three different coarse aggregates, namely, sandstone, granite and limestone, respectively, with different internal relative humidities, is determined. The initial water-to-cement mass ratio w/c is set equal to 0.40. Sandstone is used for the fine aggregates. The volume fraction of the cement paste is assumed as 0.30 and the volume fraction of the coarse aggregates with respect to the total volume of aggregates is set equal to 0.60, such that

(48) $$f_{cp}=0.30\;,\;f_{fagg}=0.28\;,\;f_{cagg}=0.42\;,$$

The thermoelastic properties of the fine and the coarse aggregates, as well as the elastic parameters of the cement paste, follow from Table 9. The thermal expansion coefficient of the cement paste follows the unsymmetrical bell-shaped function of the internal relative humidity, see Figure 1.

**Figure 6:**
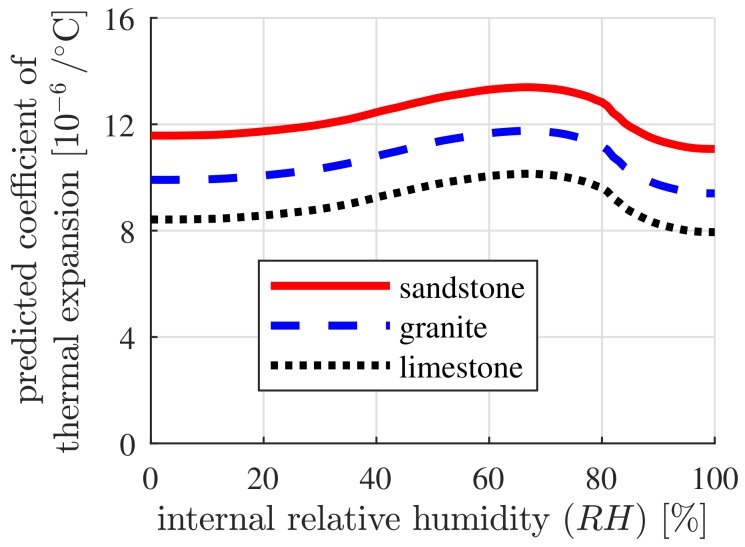
Dependence of the thermal expansion coefficient on the internal relative humidity for concrete, containing sandstone, granite and limestone, respectively, as coarse aggregates, with w/c=0.40, $f_{cp}=0.30$ and with the ratio of the volume fraction of the coarse aggregates over the total volume of aggregates equal to 0.60.

The computed thermal expansion coefficient of concrete follows an unsymmetrical bell-shaped function of the internal relative humidity, see Figure 6. The thermal expansion coefficients of fully-dried and fully-saturated concrete are almost equal, that is, they are smaller than those of partially-saturated concrete. The maximum value of the thermal expansion coefficient occurs for a concrete with an internal relative humidity of approximately 65%. Depending on the type of the coarse aggregates, the maximum variation of the thermal expansion coefficient, induced by internal relative humidity, is between 21% and 28%, see Figure 6.

### 4.3. Sensitivity of the Thermal Microstresses of Concrete with Respect to the Volumetric Composition of Concrete

In order to study the influence of the volumetric composition and the internal relative humidity on the thermal microstresses, a concrete volume is considered to be subjected to a uniform temperature change ΔT, while being macroscopically kept stress-free, that is,

(49) $$\sum_{con}=0\;,$$

this leads to an isotropic strain state of the material volume. Recalling Equation (25),

(50) $$E_{\mathrm{hom}}\;1=E_{\mathrm{hom}}=E_{\mathrm{hom}}^{e}=\alpha_{hom}\;\Delta T\;1\;,$$

with $E_{\mathrm{hom}}=\alpha_{\mathrm{hom}}\;\Delta T$ as the value of the principal components of the macroscopic strain tensor. Substituting Equation (50) into Equation (33) and considering Equation (19), delivers

(51) $$\varepsilon_{i}=\varepsilon_{i}\;1=\left(A_{i,vol}\;\alpha_{hom}+D_{im,vol}\;\alpha_{m}+D_{ii,vol}\;\alpha_{i}\right)\;\Delta T\;1\;,$$

with $\varepsilon_{i}$ as the value of the principal components of the microscopic strain tensor of the inclusion. Inserting Equation (51) into Equation (35) and considering isotropic elasticity, the microstress of the inclusion phase reads as

(52) $$\sigma_{i}=\sigma_{i}\;1=3\;k_{i}\;\left(A_{i,vol}\;\alpha_{hom}+D_{im,vol}\;\alpha_{m}+D_{ii,vol}\;\alpha_{i}-\alpha_{i}\right)\;\Delta T\;1\;,$$

with $\sigma_{i}$ as the value of the principal components of the microscopic stress tensor of the inclusion. Substituting the Equations (51) and (52) into the Equations (38)–(43), the components of the stress tensor of the ITZs, surrounding the inclusions, are obtained as

(53) $$\begin{array}{c} \sigma_{ITZ,rr}=\sigma_{i}\;,\\ \sigma_{ITZ,\theta \theta }=\sigma_{ITZ,\phi \phi }=\frac{4\;\mu_{ITZ}\left(3\;k_{ITZ}+\mu_{ITZ}\right)\;\varepsilon_{i}+\left(3\;k_{ITZ}-2\;\mu_{ITZ}\right)\left(2\;\mu_{ITZ}\;\varepsilon_{i}+\sigma_{i}\right)-18\;k_{ITZ}\;\mu_{ITZ}\alpha_{ITZ}\Delta T}{3\;k_{ITZ}+4\;\mu_{ITZ}}\;,\\ \sigma_{ITZ,r\theta }=\sigma_{ITZ,r\phi }=\sigma_{ITZ,\theta \phi }=0\;, \end{array}$$

They are independent of the zenith angle θ and the azimuth angle ϕ. The stress components $\sigma_{ITZ,rr}$, $\sigma_{ITZ,\theta \theta }$ and $\sigma_{ITZ,\phi \phi }$ are the three principal stress components. The principal stress component $\sigma_{ITZ,rr}$ is critical for debonding between the cement paste and the aggregates. Thus, it is the focus of the present sensitivity analyses.

Statistic results of nanoindentation are considered for quantification of the elastic properties of the ITZ. Young’s modulus of the ITZ is considered to be around 85% of that of the bulk cement paste while Poisson’s ratio of the ITZ is set equal to that of the bulk cement paste [34,42]. The thermal expansion coefficient of the ITZ is considered to be equal to that of the bulk cement paste, noting that the thermal expansion coefficient of the mature cement paste is practically independent of the initial water-to-cement mass ratio [11] and, thus, independent of the different porosities of the cement paste and the ITZ.

The thermal microstresses of concrete, containing three different coarse aggregates, namely, sandstone, granite and limestone, respectively, with different volume fractions, are investigated. The initial water-to-cement mass ratio, w/c, is set equal to 0.40. The internal relative humidity, RH, is assumed to be approximately equal to 50%, resulting in a value of the thermal expansion coefficient of cement paste equal to $18\times 10^{-6}{/}^{\circ }C$, see Figure 1. Sandstone is used for the fine aggregates. The thermoelastic properties of the cement paste, the fine aggregates and of three types of coarse aggregates, serving as input, follow from Table 9. The volume fraction of the cement paste, $f_{cp}$, is constant. Its value is 0.30. The ratio of the volume fraction of the coarse aggregates with respect to the total volume of aggregates follows from Equation (47).

**Figure 7:**
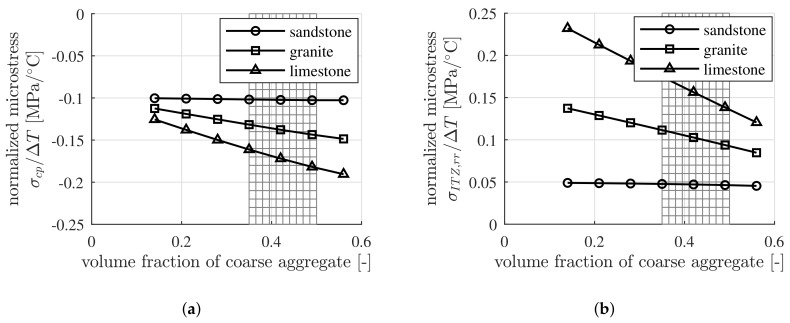
Dependence of the microstresses in (**a**) the cement paste and (**b**) the ITZs, surrounding the coarse aggregates, on the type and the volume fraction of the coarse aggregates, for concrete with RH=50%, $f_{cp}=0.30$ and w/c=0.40.

Both the volume averaged microstresses within the cement paste and the microstresses of the ITZs surrounding the coarse aggregates strongly depend on the type of the coarse aggregates, see Figure 7a,b. With increasing temperature, compressive microstresses prevail in the cement paste while tensile microstresses occur in the ITZs, surrounding the coarse aggregates. With decreasing temperature, the converse situation applies. The largest microstresses occur in the concrete that contains limestone as coarse aggregates while the smallest microstresses occur in the concrete that contains sandstone as coarse aggregates. For adequate consolidation of the concrete, the volume fraction of the coarse aggregates generally is in the range of 35% to 50% [41], see the shaded area in Figure 7a,b.

### 4.4. Sensitivity of the Thermal Microstresses of Concrete with Respect to the Internal Relative Humidity

The thermal microstresses of concrete, containing three different coarse aggregates, namely, sandstone, granite and limestone, respectively, with different internal relative humidities, are investigated. The initial water-to-cement mass ratio is set equal to w/c=0.40. Sandstone is used for the fine aggregates. The volume fractions of the concrete constituents, namely, cement paste, fine aggregates and coarse aggregates, follow from Equation (48). The thermoelastic properties of the fine and the coarse aggregates, as well as the elastic parameters of the cement paste, follow from Table 9. The thermal expansion coefficient of the cement paste follows the unsymmetrical bell-shaped function of the internal relative humidity, see Figure 1.

**Figure 8:**
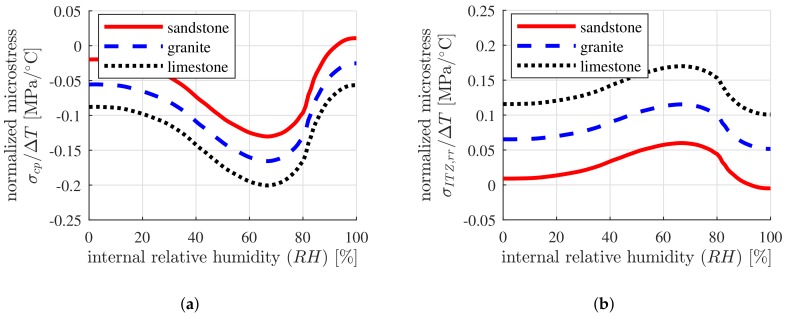
Dependence of the microstresses in (**a**) the cement paste and (**b**) the ITZs, surrounding the coarse aggregates, on the internal relative humidity of concrete containing sandstone, granite and limestone, respectively, as coarse aggregates, with w/c=0.40, $f_{cp}=0.30$ and a ratio of the volume fraction of the coarse aggregates over the total volume of aggregates equal to 0.60.

The microscopic stresses in the cement paste and the ITZs surrounding the coarse aggregates strongly depend on the internal relative humidity. They follow the unsymmetrical bell-shaped functions, see Figure 8a,b. The largest microstresses are observed for concrete with an internal relative humidity of approximately 65%, as the mismatch of the thermal expansion coefficients of the cement paste and aggregates is the largest.

## 5. Conclusions

A thermoelastic multiscale model of concrete with two types of scale transitions was presented. Bottom-up homogenization allows for quantification of the macroscopic thermal expansion and of the elastic stiffness of concrete, based on knowledge of the properties of its microstructural constituents. Conversely, top-down concentration gives access to the microstresses of the cement paste and the aggregates and furthermore, to the stress states of the interfacial transition zones covering the aggregates.

The model predictions were compared with experimental measurements by Sayki-Bekoe [2], Tasneem et al. [3] and Naik et al. [37] on concretes with different initial compositions. The established model is based on the thermoelastic constants of the cement paste and the aggregates. The model was shown to be able to predict the thermal expansion coefficients of fully-saturated concrete with acceptable accuracy, see the Table 4, Table 6 and Table 8, as well as Figure 4. This was the rationale for using the model for sensitivity analyses.

The model provides quantitative insight into the sensitivity, regarding the thermal expansion coefficient and the thermal microstresses, of concrete with respect to the composition and the internal relative humidity of the material. This leads to the following conclusions:

- Concrete consisting of siliceous aggregates exhibits a larger thermal expansion coefficient and smaller microscopic thermal stresses, compared to concrete consisting of calcareous aggregates. Both the thermal expansion coefficient and the microstresses nearly depend linearly on the volume fraction of the coarse aggregates. This agrees with qualitative findings from experimental testing [4,5,6,7].
- Concrete under partially-saturated conditions exhibits a larger thermal expansion coefficient and much larger microscopic thermal stresses, compared to concrete under fully-saturated and fully-dried conditions. This follows from the mismatch of the thermal expansion coefficients of the aggregates and the cement paste, which is an unsymmetrical bell-shaped function of the internal relative humidity.
- Temperature changes lead to microstructural stresses within concrete, even for macroscopically stress-free concrete volumes. The inhomogeneous thermal expansion coefficients of the constituents of concrete result in the incompatible thermal eigenstrain fields. The compatibility of the total strain fields requires microscopic mechanical strain fields.
- The thermal expansion coefficient of the cement paste is generally larger than that of the aggregates. In case of cooling, this leads to microscopic tensile stresses in the bulk cement paste and to compressive stresses in the aggregates and the surrounding ITZs. In case of heating, microscopic tensile stresses occur in the aggregates and the surrounding ITZs while compressive stresses occur in the bulk cement paste.

Cracking of the cement paste, induced by tensile stresses, is a serious threat for the long-term durability of concrete structures. Both a decrease and an increase of the temperature can result in microscopic tensile stresses, either in the bulk cement paste or in the ITZs, that is, thin layers of porous cement paste surrounding the aggregates. In order to reduce the magnitudes of these tensile stresses, it is recommended to reduce the mismatch of the thermal expansion coefficients of the cement paste and of the aggregates. This can be achieved

- by using aggregates with large thermal expansion coefficients, which is, however, a trade-off between increasing the thermal expansion coefficient of concrete and decreasing the microscopic thermal stresses and
- by moistening the concrete, such that the thermal expansion coefficient of the cement paste decreases.

The presented thermoelastic multiscale model for mature concrete is useful for the frequently encountered problem that the temperature changes rather quickly such that there is no significant interplay between thermal stresses and strains, on the one hand, and the viscoelastic behavior of concrete, drying and hydration of the material, on the other hand. In the future, the model will be extended to account for such couplings, which are particularly challenging at early material age. Because the chemical reaction between water and the cementitious binder is exothermal, initial hardening of concrete takes place at an increased temperature level. After the main peak of hydration is passed, the young concrete cools down. According to the coefficient of thermal expansion, cooling results in a reduction of the volume of concrete. This effect is amplified by autogenous shrinkage and eventually also by drying shrinkage. In reinforced concrete structures, the resulting overall shrinkage of concrete is constrained, resulting in tensile stresses. Creep of concrete reduces these stresses to some extent. Nevertheless, if they reach the tensile strength of concrete, early age cracking will occur. Mitigation of this problem is the topic of ongoing research [43,44]. Future pertinent multiscale models shall be based on results obtained from innovative early-age testing of the coefficient of thermal expansion [45,46,47], shrinkage [48,49] and creep [50,51,52] of cementitious materials and on corresponding multiscale early-age models for the thermal expansion [53], shrinkage [54] and creep [55,56,57] of cementitious materials. A 16% variation of the thermal expansion coefficient of concrete is expected to result in an increase of the early-age cracking risk by 15%, see Reference [58].

## Figures and Tables

**Table 1:** Elastic moduli of mature cement pastes with different initial water-to-cement mass ratios at the age of 56 days after mixing [38].

w/c [-]	0.25	0.30	0.35	0.40	0.45	0.50	0.55	0.60
**elastic modulus [GPa]**	33.7	30.3	26.9	23.6	21.8	19.4	18.1	17.2

**Table 2:** Thermal expansion coefficients [1] and elastic parameters [39] of commonly-used concrete aggregates.

Aggregate Type	Thermal Expansion Coefficient [$10^{-6}{/}^{\circ }C$]	Elastic Modulus [GPa]	Poisson’s Ratio [-]
limestone	4.75	45	0.270
dolomite	8.50	55	0.225
sandstone	11.25	35	0.250
basalt	6.75	70	0.220
diabase	6.75	78	0.215
granite	7.50	35	0.230
marble	5.50	65	0.245
quartzite	11.75	50	0.160

**Table:** (**a**)

w/c	$f_{\mathrm{cp}}$	$f_{\mathrm{fagg}}/f_{\mathrm{cagg}}$	$f_{\mathrm{fagg}}$	$f_{\mathrm{cagg}}$
0.32	0.35	40/60	0.260	0.390
		45/55	0.2925	0.3575
		50/50	0.325	0.325
0.38	0.33	40/60	0.268	0.402
		45/55	0.3015	0.3685
		50/50	0.335	0.335
0.44	0.31	40/60	0.276	0.414
		45/55	0.3105	0.3795
		50/50	0.345	0.345

**Table:** (**b**)

		Thermal Expansion Coefficient [$10^{-6}{/}^{\circ }C$]	Elastic Modulus [GPa]	Poisson’s Ratio [-]
cement paste	w/c=0.32	10.50	28.94	0.20
	w/c=0.38	10.50	24.92	0.20
	w/c=0.44	10.50	22.16	0.20
siliceous sand (quartzite)		11.75	50.00	0.16
granite		7.50	35.00	0.23
siliceous river gravel (sandstone)		11.25	35.00	0.25
dolomitic limestone (dolomite)		8.50	55.00	0.225

**Table 4:** Comparison of the model predicted with the experimentally measured thermal expansion coefficients by Sakyi-Bekoe [2] for concretes with granite, siliceous river gravel and dolomitic limestone as coarse aggregates (unit: $10^{-6}{/}^{\circ }C$).

w/c	$f_{\mathrm{fagg}}/f_{\mathrm{cagg}}$	Granite		Siliceous River Gravel		Dolomitic Limestone	
		Measured	Predicted	Measured	Predicted	Measured	Predicted
0.32	40/60	10.15	9.64	12.62	11.16	10.19	9.25
	45/55	10.24	9.79	12.28	11.17	9.56	10.04
	50/50	10.64	9.93	12.49	11.19	9.74	10.15
0.38	40/60	9.86	9.59	12.73	11.19	10.06	9.90
	45/55	9.94	9.74	12.47	11.20	10.10	10.02
	50/50	10.35	9.90	12.28	11.22	9.97	10.13
0.44	40/60	9.67	9.55	13.01	11.21	9.94	9.87
	45/55	10.03	9.71	12.35	11.23	9.81	9.99
	50/50	9.85	9.87	12.37	11.25	10.12	10.12

**Table 5:** Input for model predictions of the experiments by Tasneem et al. [3]: thermal expansion coefficients and elastic parameters of the cement paste, the fine aggregates and the coarse aggregates.

	Cement Paste	Manufactured Sand (Granite)	Natural Sand (Sandstone)	Granite	Dolomite
thermal expansion coefficient [$10^{-6}{/}^{\circ }C$]	10.50	7.50	11.25	7.50	8.50
elastic modulus [GPa]	19.40	35	35	35	55
Poisson’s ratio [-]	0.20	0.23	0.25	0.23	0.225

**Table 6:** Comparison of the model predicted with the experimentally measured thermal expansion coefficients by Tasneem et al. [3] for concretes with different volumetric compositions (unit: $10^{-6}{/}^{\circ }C$).

Coarse Aggregate	Fine Aggregate	$f_{\mathrm{cp}}$	$f_{\mathrm{fagg}}$	$f_{\mathrm{cagg}}$	Measured	Predicted
granite	manufactured sand	0.33	0.20	0.47	7.99	8.30
	natural sand	0.33	0.20	0.47	8.82	9.12
	manufactured sand	0.33	0.41	0.26	8.26	8.30
	natural sand	0.33	0.41	0.26	9.76	9.98
dolomite	manufactured sand	0.33	0.22	0.45	8.48	8.77
	natural sand	0.33	0.22	0.45	9.07	9.61
	manufactured sand	0.33	0.43	0.24	8.32	8.57
	natural sand	0.33	0.43	0.24	9.61	9.36

**Table 7:** Input for model predictions of the experiments by Naik et al. [37]: thermal expansion coefficients and elastic parameters of the cement paste, the fine aggregates and the coarse aggregates.

	Cement Paste	Natural Sand(Sandstone)	Glacial Gravel(Sandstone)	Dolomite	Quartzite	Diabase	Basalt
thermal expansion coefficient [$10^{-6}{/}^{\circ }C$]	10.50	11.25	11.25	8.5	11.75	6.75	6.75
elastic odulus [GPa]	23.6	35	35	55	50	78	70
Poisson’s ratio [-]	0.20	0.25	0.25	0.225	0.16	0.215	0.22

**Table 8:** Comparison of the model predicted with the experimentally measured thermal expansion coefficients by Naik et al. [37] for concretes with different types of coarse aggregates (unit: $10^{-6}{/}^{\circ }C$).

Coarse Aggregate	Glacial Gravel	Dolomite	Quartzite	Granite	Diabase	Basalt
measured	10.20	10.60	12.20	9.50	9.30	9.30
predicted	11.06	9.65	11.31	9.27	8.66	8.68

**Table 9:** Input for sensitivity analysis: thermal expansion coefficient and elastic parameters of the cement paste, the fine aggregates and the coarse aggregates.

	Cement Paste	Fine Aggregates	Coarse Aggregates		
		Sandstone	Sandstone	Granite	Limestone
thermal expansion coefficient [$10^{-6}{/}^{\circ }C$]	18.0	11.25	11.25	7.50	4.75
elastic modulus [GPa]	23.6	35.0	35.0	35.0	45.0
Poisson’s ratio [-]	0.20	0.25	0.25	0.23	0.27
